# Intraperitoneal oxygen microbubble therapy: A novel approach to enhance systemic oxygenation in a smoke inhalation model of acute hypoxic respiratory failure

**DOI:** 10.1016/j.sopen.2023.09.020

**Published:** 2023-10-11

**Authors:** Premila D. Leiphrakpam, Hannah R. Weber, Kirk W. Foster, Keely L. Buesing

**Affiliations:** a University of Nebraska Medical Center College of Medicine, Department of Surgery, Omaha, NE, USA; b University of Nebraska Medical Center College of Medicine, Department of Pathology and Microbiology, Omaha, NE, USA

**Keywords:** Acute respiratory distress syndrome (ARDS), Acute hypoxic respiratory failure (AHRF), Oxygen microbubbles (OMB), Extrapulmonary oxygenation, Refractory hypoxemia, Systemic oxygen augmentation

## Abstract

**Background:**

Patients suffering from severe acute respiratory distress syndrome (ARDS) face limited therapeutic options and alarmingly high mortality rates. Refractory hypoxemia, a hallmark of ARDS, often necessitates invasive and high-risk treatments. Oxygen microbubbles (OMB) present a promising approach for extrapulmonary oxygenation, potentially augmenting systemic oxygen levels without exposing patients to significant risks.

**Methods:**

Rats with severe, acute hypoxemia secondary to wood smoke inhalation (SI) received intraperitoneal (IP) bolus injections of escalating weight-by-volume (BW/V) OMB doses or normal saline to determine optimal dosage and treatment efficacy. Subsequently, a 10 % BW/V OMB bolus or saline was administered to a group of SI rats and a control group of healthy rats (SHAM). Imaging, vital signs, and laboratory studies were compared at baseline, post-smoke inhalation, and post-treatment. Histological examination and lung tissue wet/dry weight ratios were assessed at study conclusion.

**Results:**

Treatment with various OMB doses in SI-induced acute hypoxemia revealed that a 10 % BW/V OMB dose significantly augmented systemic oxygen levels while minimizing dose volume. The second set of studies demonstrated a significant increase in partial pressure of arterial oxygen (PaO2) and normalization of heart rate with OMB treatment in the SI group compared to saline treatment or control group treatment.

**Conclusions:**

This study highlights the successful augmentation of systemic oxygenation following OMB treatment in a small animal model of severe hypoxemia. OMB therapy emerges as a novel and promising treatment modality with immense translational potential for oxygenation support in acute care settings.

## Introduction

Acute respiratory distress syndrome (ARDS) poses a significant clinical challenge and remains a primary cause of morbidity and mortality in critically ill patients, with reported mortality rates between 35 and 46 % [[1], [2], [3]]. ARDS is characterized by bilateral, diffuse pulmonary infiltrates and the acute development of non-cardiogenic pulmonary edema, resulting in reduced respiratory system compliance [[4], [5], [6]]. Since its initial description by Drs. Ashbaugh and Perry in 1967 [7], extensive research has been dedicated to understanding ARDS pathophysiology and improving its management. Notably, the adoption of lung protective low tidal volume ventilation strategies, as emphasized by the ARDSNet trial [8], and advancements in extracorporeal membrane oxygenation (ECMO) have contributed to better patient outcomes. Nevertheless, a significant reduction in overall ARDS patient mortality remains an unmet need, emphasizing the importance of continued investigation and innovation. In this context, exploring novel therapeutic approaches and refining existing management strategies are crucial to further enhance the treatment and care of patients suffering from this life-threatening condition.

Severe hypoxemia, a defining feature of ARDS and acute respiratory failure, results from diverse pathological processes. Currently, few treatment modalities exist for hypoxemia unresponsive to non-invasive therapy. Mechanical ventilation is the primary invasive strategy to address respiratory failure, but it can induce complications such as ventilator-induced lung injury and hemodynamic alterations, which can trigger systemic inflammatory responses and contribute to multi-organ failure and death [9,10].

ECMO, often employed as a last resort for profound hypoxemia, bypasses the lungs and circulates the patient's blood through an external circuit to facilitate oxygen and carbon dioxide exchange [11,12]. Although it effectively corrects acute hypoxemia in the short term, 30- and 60-day adult mortality rates on ECMO remain alarmingly high, ranging from 39 to 50 % [11]. ECMO also presents numerous and significant risks, including but not limited to hemorrhage, thrombocytopenia, circuit failure, embolism, hemolysis, and limb ischemia [12,13]. Additionally, the complications linked to therapeutic-level anticoagulant use in ECMO circuits render it contraindicated for several patient populations. For instance, trauma patients with acute respiratory failure often present with traumatic brain injury, liver or spleen lacerations, or other injuries that would be exacerbated or rendered fatal if treated with therapeutic-dose anticoagulants. As a result, there is a pressing need for extrapulmonary oxygenation strategies with more favorable risk profiles.

Oxygen microbubble (OMB) therapy was designed to enhance systemic oxygenation during acute respiratory failure. OMBs consist of an oxygen core enclosed by a phospholipid monolayer and suspended in normal saline. Diameters of the OMBs range from 1 to 10 μm, as described in prior work [14]. We have previously demonstrated that intraperitoneal (IP) OMB bolus infusion – employing the abdominal cavity for oxygen exchange across the peritoneal membrane, analogous to peritoneal dialysis – leads to a substantial increase in systemic oxygen levels and 100 % survival to a predetermined 2-hour sacrifice endpoint in a lethal small animal injury model [15,16]. In the current study, we hypothesize that IP OMB treatment will significantly elevate oxygen levels in a non-lethal small animal model of severe hypoxemia secondary to inhalational injury. This hypothesis builds on our previous research and supports OMB therapy as a potential therapeutic alternative for oxygen supplementation in critically ill patients.

## Materials and methods

### Animal preparation

All animal experiments adhered to the University of Nebraska Medical Center's Institutional Animal Care and Use Committee standards. Adult male Wistar rats aged 6–8 weeks and weighing 250–290 g (*n* = 37) were procured from Charles River Laboratories, Wilmington, MA. Carotid artery catheters were implanted by the supplier before shipment, following established protocols for repeated blood sample collection. Animals were housed individually in microisolator cages in a temperature-controlled room with a 12-hour light/dark cycle and unrestricted access to food and water. A 3-day acclimatization period followed their arrival.

### Smoke inhalation and OMB treatment

**Fig. 1:**
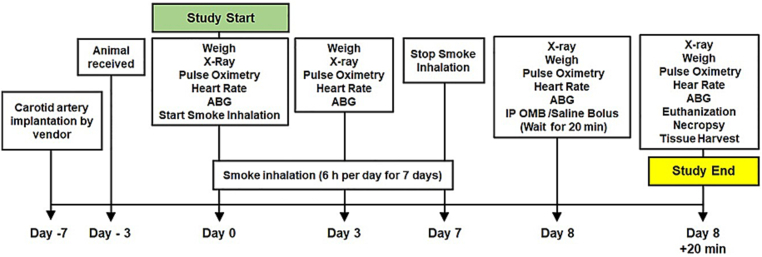
Timeline of the IP OMB treatment experiment in SI animals.

The timeline of the present study is depicted in Fig. 1; this study was conducted in two phases.

In the first phase, 18 animals were used to investigate OMB dose response following smoke inhalation. Our smoke inhalation injury model was described in detail previously [17]. Briefly, all animals were exposed to Douglas fir wood smoke for 5–6 h daily for 7 consecutive days or until the onset of acute respiratory failure, as evidenced by a sustained decrease of at least 10 % in peripheral oxygen saturation (SpO2) and the presence of bilateral, diffuse infiltrates on chest x-ray. On the 7th day of smoke exposure, animals received OMB treatment via an intraperitoneal (IP) catheter at different volumes based on their weight (body weight by volume, or BW/V): 5 % BW/V (*n* = 3), 10 % BW/V (*n* = 6), 20 % BW/V (n = 3), 40 % BW/V (n = 3), or IP normal saline bolus infusion of 10 % BW/V as a control (n = 3).

In the second phase, 19 animals were used to study the response to 10 % BW/V OMB treatment after smoke inhalation. These animals were divided into two groups, designated “SI” for those who underwent smoke exposure (*n* = 14) or “SHAM” for control animals that did not receive any smoke exposure (*n* = 5). The two groups were handled similarly throughout the study, with the exception of SHAM animals remaining in their housing room instead of undergoing smoke exposure. On the 7th day of the study, SI animals were administered a dose of 10 % BW/V OMB via an IP catheter (SI + OMB group, *n* = 11) or an equal volume of IP sterile saline (SI + Saline group, *n* = 3). Healthy SHAM animals received IP OMB (n = 3) or IP sterile saline (*n* = 2) at the same volume as the SI animals.

In both studies, SpO2, heart rate (HR), and arterial blood gas (ABG) were measured immediately before and 20 min after IP infusion. We chose the 20 min post-infusion time point based on observations from our prior work, in which treatment effect was seen at 10–20 min post-OMB infusion. After sacrifice, lung tissue was collected for wet/dry ratio and histologic scoring analysis.

### Hemodynamic monitoring

Heart rate (HR) and peripheral oxygen saturation (SpO2) were monitored using the Kent Physiosuite® (Kent Scientific Corporation, Torrington, CT) throughout the study.

Peripheral oxygen saturation (SpO2) represents the percentage of oxygenated hemoglobin compared to the total amount of hemoglobin in the blood; SpO2 is as an indirect measurement of oxygen saturation in the body and the range between 95 and 100 % is considered normal in healthy individuals [18]. A sustained decrease in SpO2 level by at least 10 % after smoke inhalation was used as an initial indicator to signal development of acute respiratory failure.

### Arterial blood gas analysis

A 300 μl arterial blood sample was drawn from the carotid artery catheter port with a syringe containing heparinized saline, following the vendor's protocol. Blood gas analysis was immediately conducted using an i-STAT VetScan Handheld Analyzer (Abaxis, Union City, CA). The following parameters were recorded: partial pressure of arterial oxygen (PaO2), partial pressure of arterial carbon dioxide (PaCO2), hydrogen bicarbonate (HCO3), pH, arterial oxygen saturation (SaO2), anion gap, base excess/deficit, hematocrit (Hct), hemoglobin (Hb), sodium (Na), ionized calcium (iCa), potassium (K), chloride (Cl), and glucose (Gluc).

### Imaging

Chest x-rays were taken for animals in both dorsoventral and lateral views immediately before and after smoke inhalation, and immediately following OMB or saline treatment. The presence of diffuse, bilateral pulmonary infiltrates on chest x-ray was used to confirm pulmonary compromise due to smoke inhalation in our animal model.

### Statistical analysis

Statistical analysis was carried out using GraphPad Prism 8 (GraphPad Software, San Diego, CA). All results were presented as the mean ± SD. One-way analysis of variance (ANOVA) with Tukey's post-hoc analysis and Student's *t*-test were employed to generate adjusted “p” values. A *p*-value <0.05 was deemed statistically significant.

## Results

### OMB dose response study in smoke inhalation-induced hypoxemia

**Fig. 2:**
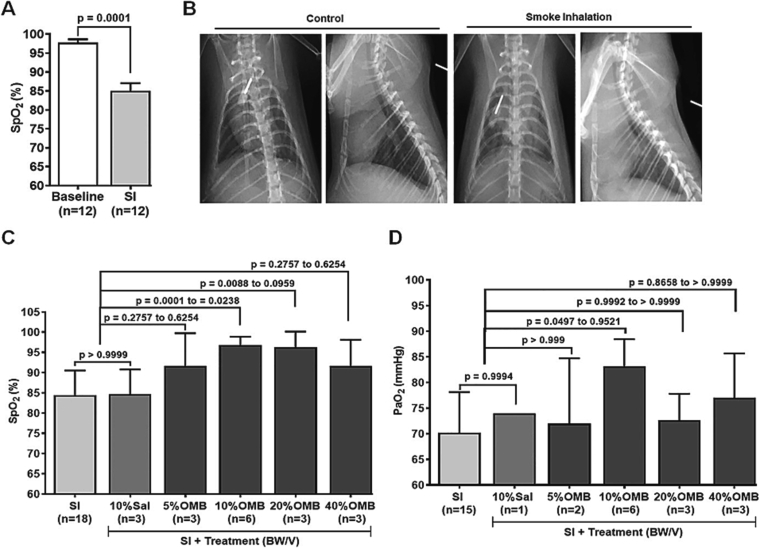
OMB dose response study in smoke inhalation-induced hypoxemia. A, SpO2 level measurement and statistical analysis at baseline and after smoke inhalation. B, Ventral-dorsal and lateral view chest x-rays taken at baseline and post SI. C, Statistical analysis of SpO2 levels at post SI and following IP injection of different doses of OMB or saline treatment. D, Statistical analysis of PaO2 levels at post SI and after IP injection of different doses of OMB or saline treatment. A *p* value of <0.05 is considered statistically significant. OMB, oxygen microbubble; SpO2, peripheral oxygen saturation; SI, smoke inhalation; IP, intraperitoneal; PaO2, partial pressure of arterial oxygen.

Animals exposed to smoke demonstrated a 10–20 % decrease in SpO2 (Fig. 2A; baseline 97.84 ± 2.85 % vs. post-SI 85.08 ± 6.84 %, *p* = 0.0001) and developed diffuse, bilateral infiltrates on chest x-ray compared with their baseline prior to smoke exposure (Fig. 2B). Treatment of different doses of OMB according to the weight of animals showed augmentation of oxygenation in SI animals as indicated by an increase in SpO2 level by 5–12 % (Fig. 2C). Maximum and statistically significant increase in SpO2 level was observed in the 10 % BW/V OMB dose group compared to the SI and SI + Saline groups (Fig. 2C; SI, 84.44 ± 6.07 % vs. SI + 10 % OMB, 96.83 ± 2.04 %, *p* = 0.0001, and SI + 10 % Sal, 84.67 ± 6.11 % vs SI + 10 % OMB, 96.83 ± 2.04 %, *p* = 0.0238), and in 20 % BW/V OMB group compared to the SI group (Fig. 2C; SI, 84.44 ± 6.07 % vs. SI + 20 % OMB, 96.33 ± 3.79 %, *p* = 0.0088). No significant change in SpO2 was observed in the saline treatment group compared with the SI group (Fig. 2C; SI, 84.44 ± 6.07 % vs. SI + 10 % Sal, 84.67 ± 6.11 %, *p* > 0.9999).

We also observed a significant increase in the partial pressure of arterial oxygen (PaO2) in the 10 % BW/V OMB group compared to SI group (Fig. 2D; SI, 70.20 ± 7.93 mmHg vs. SI + OMB, 83.17 ± 5.27 mmHg, *p* = 0.0497). Heart rate returned to the baseline level, and hemoglobin and hematocrit levels showed marginal increase following OMB treatment when compared with saline treatment (Table 1). While there were minor changes observed in select serum chemistry values, none reached statistical significance and were deemed not of clinical relevance (Table 1). Of the escalating OMB volumes used in this part of our investigations, treatment volume corresponding to 10 % BW/V provided the most significant treatment effect at the smallest volume required. This volume was used for our remaining studies.

**Table 1:**
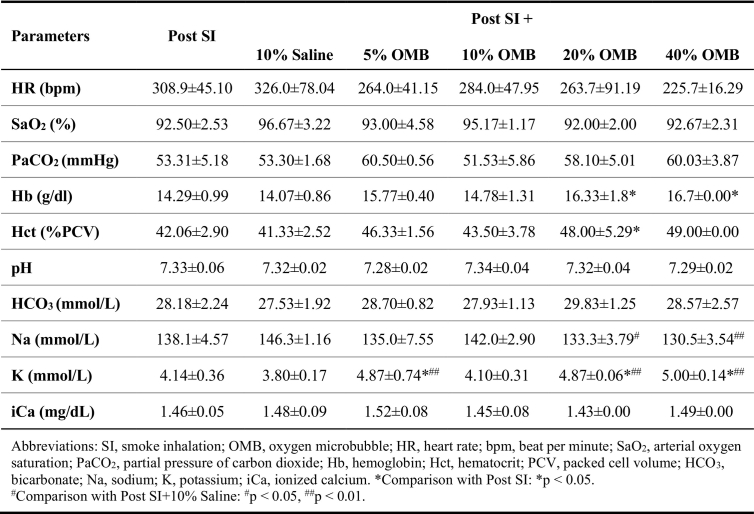
Hemodynamic and laboratory parameters after different OMB doses treatment post SI.

### OMB treatment effect on systemic oxygenation in SI-induced hypoxemia

**Fig. 3:**
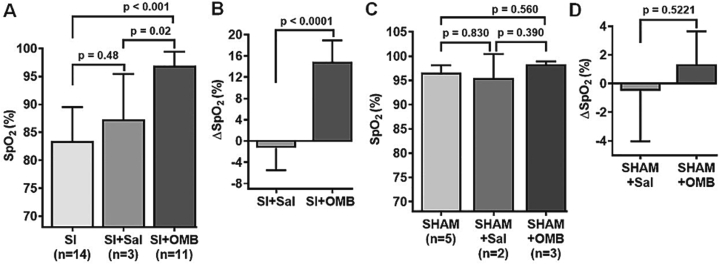
OMB treatment significantly augments SpO2 level in smoke inhalation-induced hypoxemia. A, Statistical analysis of SpO2 levels at post SI and after intraperitoneal OMB or saline treatment. B, Delta SpO2 (∆SpO2) value in the SI group with either OMB or saline treatment. C, Statistical analysis of SpO2 levels at the baseline and after intraperitoneal OMB or saline treatment in control animals, designated as SHAM. D, ∆SpO2 value in the SHAM group with either OMB or saline treatment. A *p* value of <0.05 is considered statistically significant. OMB, oxygen microbubble; SpO2, peripheral oxygen saturation; SI, smoke inhalation.

SpO2 level levels were significantly increased after IP infusion of 10 % BW/V OMB in SI animals compared with their pre-treatment levels (Fig. 3A; SI, 83.43 ± 6.10 % vs. SI + OMB, 96.91 ± 2.51 %, *p* < 0.001) and the SI + Saline treatment group (Fig. 3A; SI + 10 % Sal, 87.33 ± 8.08 % vs. SI + OMB, 96.91 ± 2.51 %, *p* = 0.02). No significant change to SpO2 was noted in SI animals who received IP saline treatment compared with their pre-treatment measurements (Fig. 3A; SI, 83.43 ± 6.10 % vs. SI + 10 % Sal, 87.33 ± 8.08 %, *p* = 0.48). A significant decrease in the delta SpO2 (ΔSpO2) level was also observed in SI + OMB group compared to SI + Saline group (Fig. 3B; SI + 10 % Sal, −1.33 ± 4.16 % vs SI + OMB, 14.91 ± 4.01 %, *p* < 0.0001). As expected, SHAM study group showed no significant changes in SpO2 or ΔSpO2 levels after either IP OMB or saline infusion (Fig. 3C and D).

**Fig. 4:**
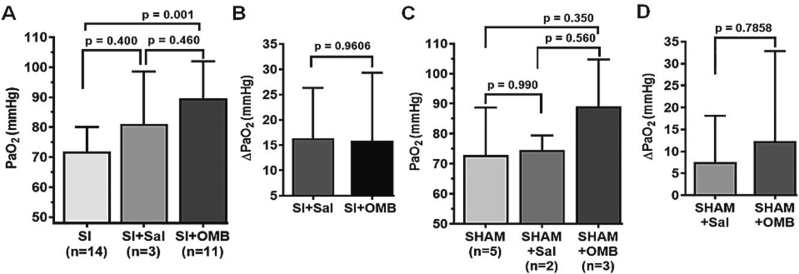
OMB treatment augments PaO2 level in smoke inhalation-induced hypoxemia. A Statistical analysis of PaO2 levels at post SI and after intraperitoneal OMB or saline treatment. B, Delta PaO2 (∆PaO2) value in the SI group with either OMB or saline treatment. C, Statistical analysis of PaO2 levels at baseline and after intraperitoneal OMB or saline treatment in control animals, designated as SHAM. D, ∆PaO2 value in the SHAM group with either OMB or saline treatment. A p value of <0.05 is considered statistically significant. OMB, oxygen microbubble; PaO2, partial pressure of arterial oxygen; SI, smoke inhalation.

PaO2 level was significantly augmented following OMB treatment post SI (Fig. 4A; SI, 71.79 ± 8.29 mmHg vs. SI + OMB, 89.64 ± 12.33 mmHg, *p* = 0.001). However, we did not observe significant change in the PaO2 or delta PaO2 (ΔPaO2) levels between the SI + OMB group and the SI + Saline group (Fig. 4A and B). In addition, no significant difference in PaO2 or ΔPaO2 levels was observed in SHAM group (Fig. 4C and D). Heart rate in SI animals trended towards normalization with OMB treatment (Table 2). Consistent with the findings in the OMB dose response experiment, we did not observe a significant change with regard to rest of the parameters measured throughout the study, although some trends can be extrapolated (Table 2).

**Table 2:**
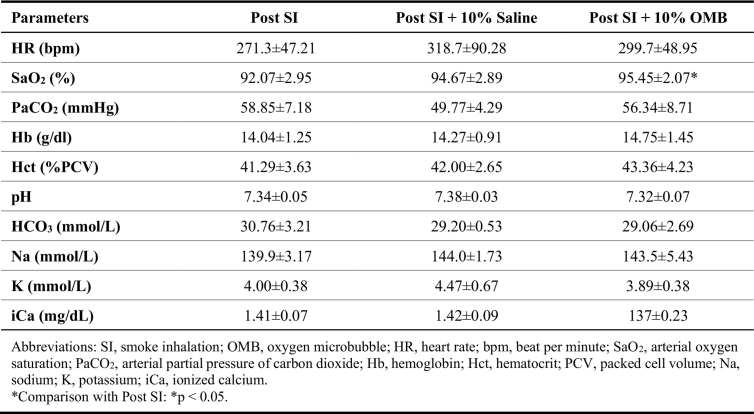
Hemodynamic and laboratory parameters after 10 % OMB treatment post SI.

**Fig. 5:**
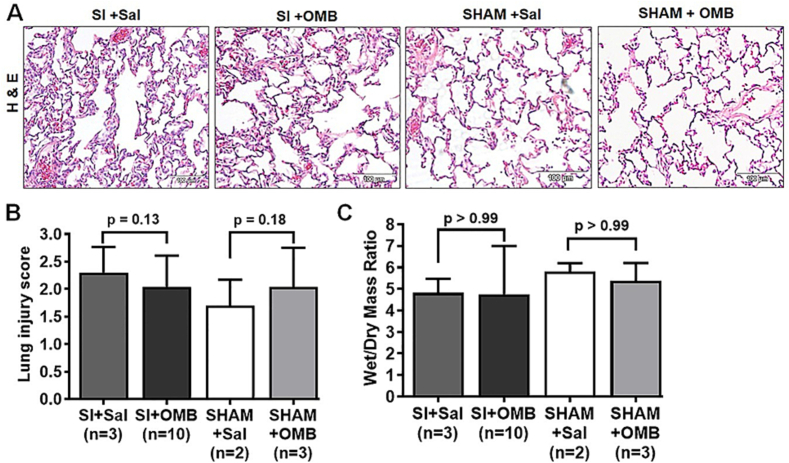
Effect of OMB treatment in lung parenchyma after smoke inhalation-induced hypoxemia. A, Statistical analysis of lung injury score between different treatment conditions in post SI and SHAM animals. B, Statistical analysis of wet/dry weight (W/D) ratio between different treatment conditions in SI and SHAM animals. A p value of <0.05 is considered statistically significant. OMB, oxygen microbubble; SI, smoke inhalation.

Previously, we have reported a significant increase in the lung injury score and lung tissue wet/dry ratio after smoke inhalation compared with healthy control animals [17]. In the current study, we did not observe significant changes in lung injury score or wet/dry ratio between the OMB and saline treated groups after smoke inhalation (Fig. 5 A and B), likely reflective that our endpoint was too acute to show significant changes in these parameters with either OMB or saline IP treatment.

## Discussion

ARDS continues to be a deadly disease, and the existing treatment options for severe, refractory hypoxemia in ARDS have not been proven to be cost-effective or to significantly reduce morbidity and mortality in patients [3]. Therefore, the development of innovative therapeutic strategies to combat hypoxemia remains crucial.

Our study builds on previous research exploring the potential of OMB therapy as a means to supplement total body oxygen levels in small animal models of acute hypoxemia. Feshitan et al. showed the effectiveness of intraperitoneal (IP) OMB therapy, achieving a 100 % survival rate in a lethal rat model of unilateral pneumothorax [15]. In another study, Legband et al. found that IP OMB treatment doubled survival time in a lethal rabbit model of complete tracheal occlusion [16].

The findings in our study demonstrate that IP OMB treatment in a small animal model of acute hypoxemia due to smoke inhalation significantly increases systemic oxygenation, as indicated by SpO2 and PaO_2_ measurements. We determined the optimal treatment dose, which resulted in the most significant effect at the lowest volume. Moreover, escalating doses of OMB were well-tolerated and caused no significant adverse effects, attesting to the safety of OMB therapy over a wide dose range. Our results indicate that IP OMB therapy is effective in treating acute hypoxemia in a small animal model of smoke inhalation, a model that more closely resembles human disease than previously studied animal models. Further studies in larger animal models are needed to confirm these findings.

The portability of OMBs makes this therapy viable in both en-route and hospital settings. Our study suggests that IP OMB therapy enhances systemic oxygen levels without many of the risks associated with other, more invasive treatment strategies. OMB therapy shows considerable potential for adaptation in human trials, and ongoing research aims to further develop OMB therapy administered through different body cavities, including the abdomen and gastrointestinal tract. The encouraging results of our study also merit further investigation into the underlying mechanisms of intraperitoneal OMB therapy. As we move towards clinical implementation, we envision OMB therapy significantly impacting not only intensive care units but also rural, remote, and military combat settings. In these situations, timely access to advanced medical care is often limited, and OMB therapy could play a crucial role in saving lives by providing rapid treatment for hypoxemia.

There were a few limitations to our study. First, this was a pilot study with an endpoint of 20 min following IP treatment of OMB or saline, performed in preparation for large animal studies. Due to the acute endpoint, no significant improvement in lung injury score or wet/dry ratio was observed in our treatment groups. As well, we did not evaluate treatment effect duration in this preliminary investigation. Additional studies with modified endpoints extended to 24 and 48 h post-treatment, as done previously [19], will address these issues. Second, our study employed a one-way delivery of oxygen without simultaneously removing carbon dioxide and might result in complications such as hypercapnia and acidosis. Our future studies in a large animal model will address these questions. Third, the peritoneal surface area of a rat does not correlate as closely with humans as larger animal models. Further research will be performed to investigate the utility of intraperitoneal microbubble oxygenation in larger animals where the ratio of peritoneal surface area to total body mass is closer to humans. Alternatively, other body cavities such as the thorax or gastrointestinal tract could also be utilized to increase the surface area for oxygen transfer.

## Conclusions

Our study represents a groundbreaking advancement in the development of oxygen microbubble (OMB) therapy as a potential treatment for acute hypoxemia. This innovative and portable therapy has the potential to revolutionize hypoxemia management in a wide range of clinical settings, extending its impact beyond what conventional treatments can offer. By addressing the unmet needs of patients with hypoxemia in various scenarios, OMB therapy has the potential to fill a critical gap in the current therapeutic landscape and significantly impact not only intensive care units but also rural, remote, and military combat settings. The promising results of our pilot study provide strong justification for further research into the effectiveness and safety of OMB therapy in larger animal models and, eventually, human trials.

## Ethics approval

All animal studies were performed in accordance with the University of Nebraska Medical Center/Nebraska Medicine (UNMC/NM; Omaha, Nebraska) Institutional Animal Care and Use Committee standards.

## Funding

This work was supported by the Department of Defense 10.13039/100006831U.S. Air Force [award number FA4600-12-D-9000].

## CRediT authorship contribution statement

Conception and design: KLB; Data collection: HRW, KLB; Analysis and interpretation: PDL, HRW, KLB, KWF; Drafting original manuscript: PDL. All authors contributed to the critical revision of the manuscript draft and approval of the final version of the manuscript.

## Declaration of competing interest

Keely L. Buesing has financial holdings in the company Respirogen, Inc. in the form of stock option agreements. Respirogen, Inc. looks to commercialize the oxygen microbubble technology.

## Data Availability

Data are available upon reasonable request from the corresponding author.
